# Do influence at work and possibilities for development mitigate the impact of job demands for workers with and without depression

**DOI:** 10.5271/sjweh.4069

**Published:** 2023-02-27

**Authors:** Patricia Ots, Anita C Keller, Eva Altrock, Sander KR van Zon, Sandra Brouwer

**Affiliations:** 1 Department of Health Sciences, Community and Occupational Medicine, University Medical Center Groningen, University of Groningen, The Netherlands; 2 Department of Psychology, University of Groningen, The Netherlands

**Keywords:** employment, job demand–control model, mental health

## Abstract

**Objective:**

Jobs characterized by low job demands and high job resources are associated with better work outcomes, yet it remains unclear whether this is the case for workers with depression. This study examined whether depression moderates the relationship between job demands, job resources, and maintaining employment.

**Methods:**

Data from the longitudinal population-based Lifelines cohort study were matched with register data on employment from Statistics Netherlands (N=55 950). Job demands included quantitative demands and work pace; job resources included influence at work and possibilities for development. The two-way interaction between job demands and depression and the three-way interaction between job demands, job resources and depression were examined in a zero-inflated Poisson regression model with path 1, including a binary employment outcome, and path 2, a count variable including months out of employment.

**Results:**

The interaction effect of job demands and depression on being employed was significant [b=-0.22, 95% confidence interval (CI) -0.44‒0.01]. Workers without depression were more likely to be employed whereas workers with depression were less likely to be employed if they had high job demands. The three-way interaction between job demands, job resources, and depression was significant for months out of employment (b=0.15, 95% CI 0.01‒0.29), indicating that workers with depression had more months out of employment when reporting high job demands and high job resources compared to workers without depression.

**Discussion:**

Although increasing influence at work and possibilities for development to prevent negative work outcomes may be beneficial for workers without depression, this approach might be limited for workers with depression.

Work is a fundamental part of adult life offering a variety of benefits such as financial stability and purpose (1, 2). Generally, having paid work is related to better physical and mental health over time compared to not having paid work (3). The importance of work is further illustrated by the detrimental and lasting effects of unemployment on well-being (4). Given that work is an important part of adult life, accessible and sustainable job opportunities are needed, especially for vulnerable groups such as workers with mental disorders (5).

In many industrialized countries mental disorders are on the rise, with depression being one of the most common mental disorders in the general population and more specifically in the workforce (5). In Europe, about 40 million people are affected by depression (6). Depression is known to have a high rate of recurrence and entails symptoms such as feelings of tiredness, poor concentration, and overall sadness, which can negatively impact daily living (7). While some studies show that work exerts benefits for workers with depression [eg, increases in self-esteem, personal growth, sense of purpose (8, 9)], studies have also shown that workers with depression are more prone to negative work outcomes such as reduced productivity at work, reduced work capacity, prolonged sickness absence, and early exit from paid employment into unemployment and disability pension (10–14).

Previous research among healthy workers has indicated that the work environment influences the likelihood of maintaining employment (15, 16). Workers perform their work under different conditions such as the degree of time pressure or control to decide how, where, and when tasks are performed. Two influential theoretical models are the Job Demand–Control (JDC) model (17) and the Job Demand–Resource model (JDR) (18), which theorize the degree of job demands and job resources to predict work and well-being outcomes. In general, job demands are aspects of the job that require effort and are associated with more negative work outcomes such as burnout and low work engagement (15, 19) while job resources are positively associated with work engagement (20). Studies have shown that job demands reduce work ability while job resources increase work ability (21). Among healthy workers, a job that is characterized by low-to-moderate job demands and high job resources is positively related to well-being and employee engagement (22, 23). Although job resources can include task resources, interpersonal resources, and leadership resources (24), we focused on task resources in the current study, which are from now on referred to as “job resources”.

While extensive research has given insight into how working conditions affect work among healthy workers, job characteristics may exert a different influence among workers with depression. Symptoms of depression may make dealing with job demands more challenging and could increase the need for resources such as job control, as these resources might support the workers’ capacity to function at work. People with depression more often use rumination, avoidance, and suppression when confronted with difficult internal or external situations (25). Rumination cannot only induce and prolong the experience of negative emotions, it can also reduce executive functioning by limiting working memory, attention, and cognitive control (26–28). Job resources such as job control can help workers with depression to navigate fluctuating symptoms by allowing them to rearrange their schedule to fit best with their mental state. In line with that idea, limited resources, such as low job control, have been associated with an increased risk of entering disability pension over an 11-year period among workers with depression (29).

The aim of the current study was to examine whether depression moderates the relationship between job demands, job resources, and maintaining employment. We aimed to provide a more nuanced view on job demands and resources by testing their functioning for workers with and without depression. In doing so, we explored potentially relevant boundary conditions of widely used theoretical propositions. Ultimately, understanding how job characteristics function for different groups in the labor market may help organizations to design work that is more inclusive.

## Method

### Participants and procedure

The current study used data from the Dutch longitudinal population-based Lifelines cohort study and biobank matched with register data from Statistics Netherlands (Centraal Bureau voor de Statistiek) (30). Lifelines is a multi-disciplinary prospective population-based cohort study examining in a unique three-generation design the health and health-related behaviors of 167 729 persons living in the north of the Netherlands. It employs a broad range of investigative procedures in assessing the biomedical, socio-demographic, behavioral, physical and psychological factors, which contribute to the health and disease of the general population, with a special focus on multi-morbidity and complex genetics. Participants were recruited via general practitioners and family members or they could self-register (31).

The study was conducted according to the guidelines in the Declaration of Helsinki and approved by the medical ethical review committee of the University Medical Center Groningen (ethics number: 2007/152). All participants provided their written informed consent. The Lifelines cohort study collected data in a number of waves. Of importance for this study are the baseline wave T0 (2006–2013) – during which depression was measured – and T2 (2012–2017) – during which job demands and job resources were measured. Data from Lifelines was linked to monthly employment status data from Statistics Netherlands for up to two years after T2. The final study sample consisted of 55 950 adult participants aged 18–64 who were employed and completed questionnaires at T2 (see supplementary material, www.sjweh.fi/article/4069, figure S1 for the sample selection and table S1 for the measurements).

### Measures

*Job demands and job resources*. Job demands and job resources were measured with questions from the Copenhagen Psychosocial Questionnaire second version (COPSOQ2) at T2 (32). All questions focused on the experiences at work in the past month. For job demands, two questions focused on quantitative demands: “Do you have enough time for the work you need to do?” (reversed) and “Do you get behind in your work?”. In addition, two questions focused on work pace: “Is the work pace high throughout the workday?” and “Do you have to work very fast?”. Each item was answered on a 5-point Likert scale [1=always to 5=(almost) never]. The Cronbach’s alpha was 0.73. The four questions on job resources were divided into two sub dimensions: influence at work and possibilities for development. Influence at work included “Do you have a high degree of influence on your work?” and “Can you influence the amount of work you have to do?”, and possibilities for development included “Do you have the possibility to learn new things through your work?” and “Does your work require you to take the initiative?”. The influence at work had answer options ranging from 1 (always) to 5 (almost) never), while the possibilities for development questions had answer options ranging from 1 (to a very high degree) to 5 (to a very small degree). According to the confirmatory factor analysis (CFA), the second item did not load onto the latent factor of job resources and was therefore excluded. The Cronbach’s alpha of the three remaining items was 0.61. For the analyses, mean scores were used for job demands and job resources. Both job demands and job resources were centered. Working conditions were recoded so that higher values represented more demands and resources respectively.

### Employment status

Register data from Statistics Netherlands was matched with the data from Lifelines to track the employment status. Employment status at T2 was included for every month in the following two years. Participants were classified as having employment when their main income component was from paid employment (excluding self-employed). The following categories were counted as not having paid employment: receiving disability benefits or unemployment benefits, early retirement, and economic inactivity. Employment status was divided in two parts. Employment status was classified as “being in paid employment” (0) and “not being in paid employment” (1) per month. The variable “months out of paid employment” reflected the number of months not having employment during the two years after T2, ranging from 0 to 23.

### Depression at baseline

Depression was assessed at baseline (T0) using the Mini-International Neuropsychiatric Interview (MINI) (33). The MINI is a short structured diagnostic interview developed by clinicians and psychiatrists mainly designed for research purposes. It can be used to assess the diagnosis of psychiatric patients using the ICD-10 and DSM-IV criteria. In this study, participants were classified as having depression at baseline if (i) they had a depressive disorder or (ii) they had a dysthymic disorder and used antidepressants (ATC-code N06A) as reported by trained research staff. For the remainder of the paper, we refer to “depression” to indicate “depression at baseline”.

### Sociodemographic and health characteristics

Sociodemographic characteristics were assessed at T2 and included age, gender, and educational level. Educational level was categorized into low (no education, primary education, lower or preparatory vocational education, junior general secondary education), intermediate (secondary vocational education or work-based learning, senior general secondary education or pre-university secondary education), high (higher vocational education, university) educational degree. Perceived health was assessed using the one-item short-form survey (SF-1) of the original SF-12 questionnaire: “In general, would you say that your health is ‘excellent’, ‘very good’, ‘good’, ‘fair’, or ‘poor’?” (34).

### Statistical analysis

First, descriptive statistics of the sample were examined. Second, we performed a confirmatory factor analysis on the latent constructs of job demands and job resources to assure empirical distinctiveness. Third, a Poisson regression baseline model and a zero-inflated Poisson (ZIP) regression baseline model were estimated. The ZIP regression runs one model with two regression coefficients (35). It distinguishes between two outcomes: first, the binary outcome of being employed (yes/no) throughout the follow-up period and, second, the count variable months out of employment. Job demands and job resources were included as continuous measures. The exponential function of the coefficients indicates how the odds of being in paid employment [odds ratio (OR)] and the predicted months out of employment [incident risk ratio (IRR)] respectively change when job demands and job resources change by one point. The regression coefficient for months out of employment focusses only on participants that had at least one month out of employment during follow-up. We examined five models. Model 1 included the main effects of job demands, job resources and depression. Model 2 included the two-way interaction between job demands and job resources. In model 3 and 4, the two-way interactions between job demands and depression and between job resources and depression were assessed. Lastly, in model 5, the three-way interaction between job demands, job resources and depression was examined. Simple slopes comparing workers with and without depression were examined with a slope-difference test. Predicted values of the unadjusted models were plotted for an interpretation of the moderation effect (36). Low and high job resources were defined by the mean score ±1 SD. Model fit was assessed by a log-likelihood ratio test, with smaller values indicating a better model fit (37). In addition, we assessed model fit relying on the root mean squared error of approximation (RMSEA), standardized root mean square residual (SRMR), and comparative fit index (CFI). An adequate model fit is indicated by a RMSEA value <0.08 (38), while a good model fit is indicated by a SRMR value >0.05 (39) and CFI ≥0.95 (37). Models were built up adjusting for the covariates. Workers who reached the statutory retirement age of 65 during the two-year follow-up period were excluded from the analyses as well as workers with missing values on the working conditions or covariates. All analyses were performed in Mplus 8.0 with a robust maximum likelihood estimator (MLR).

## Results

The mean age of the study population was 44.4 (SD 9.8) years and 59.7% was female. Of all workers, 21.8% had a low, 41.3% had an intermediate and 35.3% had a high educational level. On average, workers rated their health as good to very good (mean 3.3, SD 0.8). The mean score for job demands was 2.8 (SD 0.7) and for job resources 3.4 (SD 0.7). Most workers (87.5%) remained employed throughout the two-year follow-up period. From the total sample, 1188 (2.1%) of the workers had depression. In total, 87.7% of workers without depression and 81.0% of workers with depression remained employed during follow-up. For workers who had ≥1 month out of employment, workers without and with depression had an average of 7.9 (SD 6.3) and 9.8 (SD 6.7) months out of employment, respectively. At T2, sociodemographic characteristics, perceived health and job demands and job resources did not differ between workers with and without depression. For the final analyses, 240 workers were excluded due to reaching the statutory retirement age of 65 (table 1). In addition, 2435 (4.4%) workers were excluded due to missing data on working conditions and 147 (0.3%) due to missing data on covariates.

**Table 1:** Descriptive statistics of the study sample. [SD=standard deviation.]

	Total sample (N=55 950)		No depression (N=54 762)		Depression (N=1188)	
		
	Mean (SD)	%	Mean (SD)	%	Mean (SD)	%
Age in years	44.4 (9.8)		44.4 (9.8)		44.1 (9.5)	
Gender						
Male		40.3		40.6		29.8
Female		59.7		59.4		70.2
Educational level						
Low		21.8		21.5		35.8
Intermediate		41.4		41.4		42.6
High		35.3		35.7		20.2
Other		1.5		1.5		1.4
Perceived health (1–5) ^a^	3.3 (0.8)		3.3 (0.8)		2.8 (0.7)	
Job demands (1–5) ^a^	2.8 (0.7)		2.8 (0.7)		2.8 (0.7)	
Job resources (1–5) ^a^	3.4 (0.7)		3.4 (0.7)		3.2 (0.7)	
Employment status during follow-up						
Employed		87.5		87.7		81.0
Out of employment		12.5		12.3		19.0
Months out of employment (1–23)	8.0 (6.3)		7.9 (6.3)		9.8 (6.7)	
Reached statutory retirement age		0.4		0.4		0.1

a Job demands, job resources and perceived health were recoded, with higher scores representing more demands and resources and better health. Missing values: education 0.1%; perceived health <0.1%, job demands 4.3%: job resources 4.3%, employment: 0.8%.

We tested if job demands and job resources are two empirically distinct constructs. The CFA comparing a 1- versus 2-factor model clearly favored the 2-factor model (1-factor solution: SB-scaled χ^2^(53 531)=26 784.61, RMSEA=0.19, SRMR=0.12, CFI=0.58; 2-factor solution: SB-scaled χ^2^(53 531)=2855.32, RMSEA=0.07, SRMR=0.04, CFI=0.96). Next, Bayesian Information Criterion (BIC) values of the Poisson regression baseline model (BIC=242 798.31) and the ZIP regression baseline model (BIC=87 667.45) were compared, showing a better model fit for the ZIP regression baseline model. The log-likelihood ratio test (Poisson regression baseline model: log-likelihood=-121 371.95; ZIP regression baseline model: log-likelihood=-43 779.31; log-likelihood ratio *X^2^* (5, N=53 229)=-23 620.53, P<0.001), favored the ZIP regression baseline model as well.

### Main and interaction effects on employment and months out of employment

First, we analyzed the main effects of job demands, job resources and depression on employment and months out of employment in model 1 (log-likelihood=-42 999.56, BIC=86 173.22) (table 2). In the fully adjusted model, workers with high job demands were more likely to be employed (b=0.18, 95% CI 0.14‒0.23; OR=1.20, 95% CI 1.15‒1.26). In addition, workers who reported having high job resources were more likely to maintain employment (b=0.29, 95% CI 0.24‒0.33; OR=1.34, 95% CI 1.27‒1.39). Workers with depression were less likely to maintain employment (b=-0.31, 95% CI -0.48‒-0.15; OR=0.73, 95% CI 0.62‒0.86) and were more likely to have more months out of employment (b=0.12, 95% CI 0.02‒0.23; IRR=1.13, 95% CI 1.02‒1.26). The impact of job demands and job resources on months out of employment was only observed in the unadjusted models.

**Table 2:** Model effects for being employed (path 1) and months out of employment (path 2). Job demands and job resources were recoded, with higher scores representing more demands and resources. The simple slopes of job demands are shown for low, medium and high levels of job resources. [CI=confidence interval]

	Unadjusted		Adjusted for sociodemographic characteristics		Adjusted for sociodemographic characteristics and perceived health	
		
	Estimate	95% CI	Estimate	95% CI	Estimate	95% CI
Being employed (path 1)						
Job demands	0.20	0.15–0.24	0.17	0.12–0.21	0.18	0.14–0.23
Job resources	0.36	0.33–0.40	0.30	0.25–0.34	0.29	0.24–0.33
Depression	-0.40	-0.56– -0.23	-0.36	-0.53– -0.20	-0.31	-0.48– -0.15
Job demands×job resources	0.06	0.01–0.12	0.05	-0.00–0.11	0.05	-0.00–0.11
Job demands–low job resources	0.18	0.14–0.22	0.15	0.11–0.20	0.17	0.12–0.21
Job demands–medium job resources	0.21	0.17–0.26	0.18	0.13–0.22	0.19	0.15–0.24
Job demands–high job resources	0.24	0.18–0.30	0.21	0.15–0.26	0.22	0.16–0.28
Job demands×depression	-0.23	-0.45– -0.01	-0.20	-0.42–0.02	-0.22	-0.44–0.01
Job resources×depression	-0.12	-0.33–0.10	-0.09	-0.31–0.13	-0.10	-0.32–0.13
Months out of employment (path 2)						
Job demands	-0.03	-0.06– -0.00	-0.02	-0.04–0.01	-0.02	-0.05–0.01
Job resources	-0.04	-0.06– -0.01	-0.02	-0.05–0.01	-0.02	-0.05–0.01
Depression	0.12	0.02–0.23	0.14	0.04–0.25	0.12	0.02–0.23
Job demands×job resources	-0.04	-0.08– -0.01	-0.03	-0.06–0.00	-0.03	-0.06–0.01
Job demands–low job resources	-0.02	-0.05–0.01	-0.01	-0.04–0.02	-0.02	-0.05–0.01
Job demands–medium job resources	-0.04	-0.07– -0.01	-0.02	-0.05–0.01	-0.03	-0.06–0.00
Job demands–high job resources	-0.06	-0.09– -0.02	-0.04	-0.08– -0.00	-0.04	-0.08– -0.01
Job demands×depression	0.03	-0.09–0.16	0.03	-0.09–0.15	0.02	-0.09–0.14
Job resources×depression	-0.02	-0.14–0.10	-0.03	-0.14–0.09	-0.04	-0.16–0.08

In model 2, the interaction between job demands and job resources was added (log-likelihood=-42 991.50, BIC=86 178.85). Workers with a combination of high job demands and high job resources were more likely to be employed (b=0.05, 95% CI -0.00‒0.11; OR=1.05, 95% CI 1.00‒1.12). The simple slopes showed that job demands were only significantly associated with months out of employment when job resources were at a medium or high level, meaning that it was most likely to have less months out of employment in a job characterized by high demands and medium or high levels of job resources (table 2).

### The moderating role of depression on job demands and job resources on employment and months out of employment

**Figure 1:**
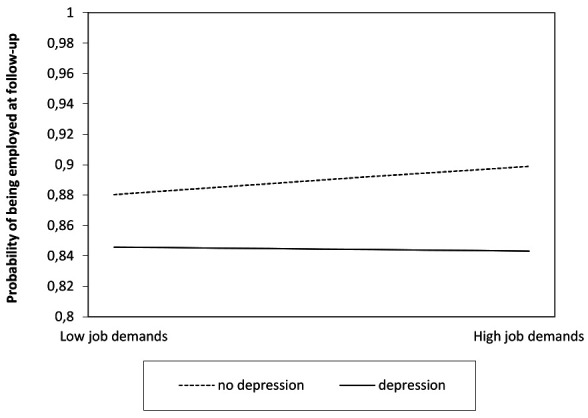
The unadjusted two-way interaction between job demands and depression: path 1 - employment.

In models 3 and 4, the two-way interaction term between job demands and depression and job resources and depression respectively were added to the model (log-likelihood=-42 997.30, BIC=86 190.46; log-likelihood=-42 998.31, BIC=86 192.47). The interaction effect of job demands and depression on being employed was significant (b=-0.22, 95% CI -0.44‒0.01; OR=0.80, 95% CI 0.64‒1.01), while the interaction of job demands and depression on months out of employment was non-significant. Job demands did not have an effect on being employed among workers with depression (b=-0.02, 95% CI -0.24‒0.20; OR=0.98, 95% CI 0.79‒1.22), whereas workers without depression were more likely to be employed if they had high job demands (b=0.21, 95% CI 0.16‒0.25; OR=1.23, 95% CI 1.17‒1.28) (table 3). The probability of being in paid employment increased from 88.0% to 89.9% for workers without depression in case of having high job demands (figure 1). The interaction between job resources and depression was non-significant.

**Table 3:** Simple slopes and slope difference tests for being employed and months out of employment in unadjusted models stratified by depression status. Job demands and job resources were recoded, with higher scores representing more demands and resources. [SDT=slope difference test; CI=confidence interval]

	No depression		Depression		SDT
		
	Estimate	95% CI	Estimate	95% CI	P-value
Being employed (path 1) ^a^					
Job demands	0.21	0.16–0.25	-0.02	-0.24–0.20	<0.05
Job resources	0.37	0.33–0.41	0.25	0.04–0.47	0.30
Job demands–low job resources	0.22	0.08–0.36	-0.02	-0.24–0.19	0.08
Job demands–medium job resources	0.22	0.17–0.26	-0.03	-0.29–0.24	0.08
Job demands–high job resources	0.22	0.08–0.35	-0.03	-0.38–0.33	0.08
Months out of employment (path 2) ^a^					
Job demands	-0.03	-0.06–0.00	0.01	-0.12– -0.13	0.61
Job resources	-0.04	-0.06–0.01	-0.06	-0.17–0.06	0.74
Job demands–low job resources	-0.02	-0.05–0.01	-0.00	-0.12–0.12	0.84
Job demands–medium job resources	-0.04	-0.07– -0.01	0.05	-0.08–0.18	0.17
Job demands–high job resources	-0.06	-0.10– -0.02	0.11	-0.06–0.28	<0.05

a The overall three-way interaction was significant for months out of employment (P=0.02) and not for being employed (P=0.64).

**Figure 2:**
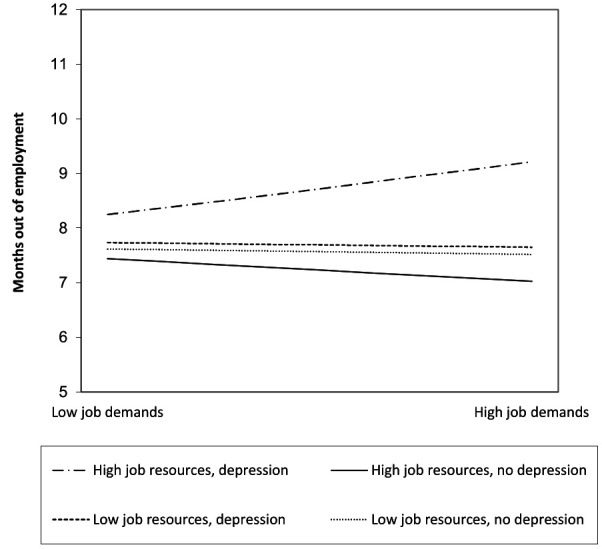
The unadjusted three-way interaction between job demands, job resources and depression: path 2 – months out of employment.

Finally, in model 5, we added the three-way interaction between job demands, job resources, and depression to the model (log-likelihood=-43 982.01, BIC=86 203.39). The three-way interaction was not significant for being employed, but was significant for months out of employment (b=0.15, 95% CI 0.01‒0.29; IRR=1.16, 95% CI 1.01‒1.34). The interaction of high job demands, high job resources and depression was related to more months out of employment during the two-year follow-up. The simple slopes show that higher job demands and high job resources only resulted in less months out of employment among workers without depression (b=-0.06, 95% CI -0.10‒-0.02; IRR=0.94, 95% CI 0.90‒1.02) and were associated with more months out of employment for workers with depression (albeit non-significantly) (table 3). Workers with depression were on average 9.2 months out of employment whereas workers without depression were on average 7.0 months out of employment in case of high job demands and high job resources (figure 2).

## Discussion

The aim of this study was to identify the differential impact of job demands and job resources on the probability of maintaining employment for workers with and without depression. To summarize, workers without depression were more likely to be employed throughout the two years follow-up if they reported high job demands. For workers with depression, job demands were not associated with employment. Moreover, if a worker experienced some months out of employment during follow-up, the period of non-employment was more than two months shorter for workers without depression if they had a combination of high job demands and high job resources. In contrast, for workers with depression, having high job demands and high job resources did not result in less months out of employment, and these workers seemed to be more likely to have more months out of employment.

The finding that job demands were associated with a higher chance of being employed during follow-up is in line with studies showing that higher job demands were associated with a lower risk of unemployment (40, 41). In these studies and in the current study, job demands were represented by workload and workpace, a job demand that is sometimes classified as a challenge stressor (42). Challenge stressors have negative implications for well-being and health, especially if exposed to over longer time periods, but they also have benefits for performance. Stressors such as workload motivate workers and offer opportunities for learning (43). While high job demands are often thought of as detrimental for well-being and health, they may also offer stimulating experiences such as mastery, learning, and recognition. However, this may not be the case for workers with depression. While high job demands were potentially perceived as stimulating and engaging for workers without depression (44), workers with depression may not reap the benefits as they are unable to cope with the more negative sides of high job demands. For them, high job demands might mainly increase strain (45).

The combination of having high job demands and high job resources also differed for workers with and without depression. While workers without depression benefited from a combination of high job demands and high job resources, which was reflected by a shorter period out of employment at follow-up, this was not the case for worker with depression. While a lot of research and practice emphasizes the importance of adding resources to prevent and combat negative work outcomes as well as strengthen good ones (19, 20), this approach might be limited for the vulnerable subgroup of workers with depression.

The results of the current study are somewhat ambivalent regarding the two outcome measures of being employed and the duration of not being employed at follow-up. While depression moderated the effect of job demands for employment, it did not moderate the effect for the months out of employment. On the contrary, depression moderated the interaction between job demands and job resources for the months out of employment at follow-up but not for the likelihood of being employed. Future research could examine whether there is a substantive theoretical difference between the concepts of being employed and the duration of non-employment for workers with depression.

### Strengths and limitations

The current study has several strengths. First, data from the Lifeline Cohort Study allowed us to study the hypotheses in a sample of over 55 000 workers. Second, register data were used for the outcome measure, which is more accurate than self-report data and eliminates non-response at follow-up. Third, depression was assessed with the MINI questionnaire, which is a valid measure used to assess the diagnosis of psychiatric disorders using the ICD-10 and DSM-IV criteria. This study also has limitations. First, we did not examine the severity of depression, which might have influenced both the direct and moderation effects of depression on maintaining employment. Second, there was a gap of about two years between T0 and T2, which could have led to misclassification of workers with and without depression at T2. Lastly, the Cronbach’s alpha between the items reflecting job demands and job resources was rather low. Additional items may be needed to measure these working conditions more accurately.

### Implications

The results of the current study have important theoretical and practical implications. They show that while workers with depression have a decreased probability to maintain employment, most workers are able to remain employed. However, in contrast to workers without depression, they benefit neither from high job demands nor from a combination of high job demands and high job resources. These results indicate that while the JDC model (17) as well as more recent models such as the JDR model (18) seem fitting to predict work outcomes in healthy populations, they may need to be refined when trying to predict work outcomes for vulnerable subgroups of workers such as those with depression. The results of the current study may have implications for the design of more inclusive work. While earlier research often encourages employers to simply increase job resources to balance job demands in an effort to achieve better work outcomes (eg, job performance, attendance), the current study shows that for workers with depression there might be limits to this “one size fits all” approach.

In this study, it was argued that cognitive limitations and maladaptive emotion regulation strategies could explain why workers with depression struggle more with job demands compared to non-depressed workers. Future studies could examine such underlying mechanisms, which was outside of the scope of this study. While this study focused on job resources only, future research should investigate if other resources can support the challenges of job demands on maintaining employment as experienced by workers with depression. One resource that deserves consideration is social support as it might be an especially valuable and necessary resource for somebody with depression. Instrumental social support at work could be beneficial in dealing with high workload (46).

In addition, while the current study focused on baseline prevalence of depression, future studies could examine whether the role of job demands and job resources differs for incident cases of clinical depression or lifetime depression. In the current study, we used a clinical diagnosis of depression at T0. Therefore, our sample may have included workers with chronic, remittent and prior depression. Our results indicate that depression can have longstanding effects on employment.

### Concluding remarks

Our results show that while job demands are associated with a higher likelihood of employment during follow-up for workers without depression, this is not the case for workers with depression. Furthermore, we found that higher job resources did not help workers with depression if they had high job demands. This indicates that workers with depression do not benefit from high job demands nor from the combination of high job demands and high job resources. The effect of adding job resources to prevent and combat negative work outcomes as well as strengthen good ones might be limited for workers with depression.

## Supplementary material

Supplementary material
