# Supplementary material for: Do influence at work and possibilities for development mitigate the impact of job demands for workers with and without depression
Source: Scand J Work Environ Health. 2023 Feb 27;49(2):136–44. doi: 10.5271/sjweh.4069 (PMC10577017; doi:10.5271/sjweh.4069)
Supplement: Supplementary material [file SJWEH-49-136-S001.pdf]

Do influence at work and possibilities for development mitigate the impact of job demands for workers with and without depression[1]

by Patricia Ots, MSc,[2] Anita C Keller, Dr, Eva Altrock, MSc, Sander KR van Zon, Dr, Sandra Brouwer, Prof

1. Supplementary material
2. Corresponding to: Patricia Ots, Department of Health Sciences, Community and Occupational Medicine, University Medical Center Groningen, University of Groningen, Hanzeplein 1, 9700 RB Groningen, The Netherlands. [E-mail: p.ots@umcg.nl]

Supplementary figure S1. Flow chart of inclusion in the Lifelines Cohort Study.

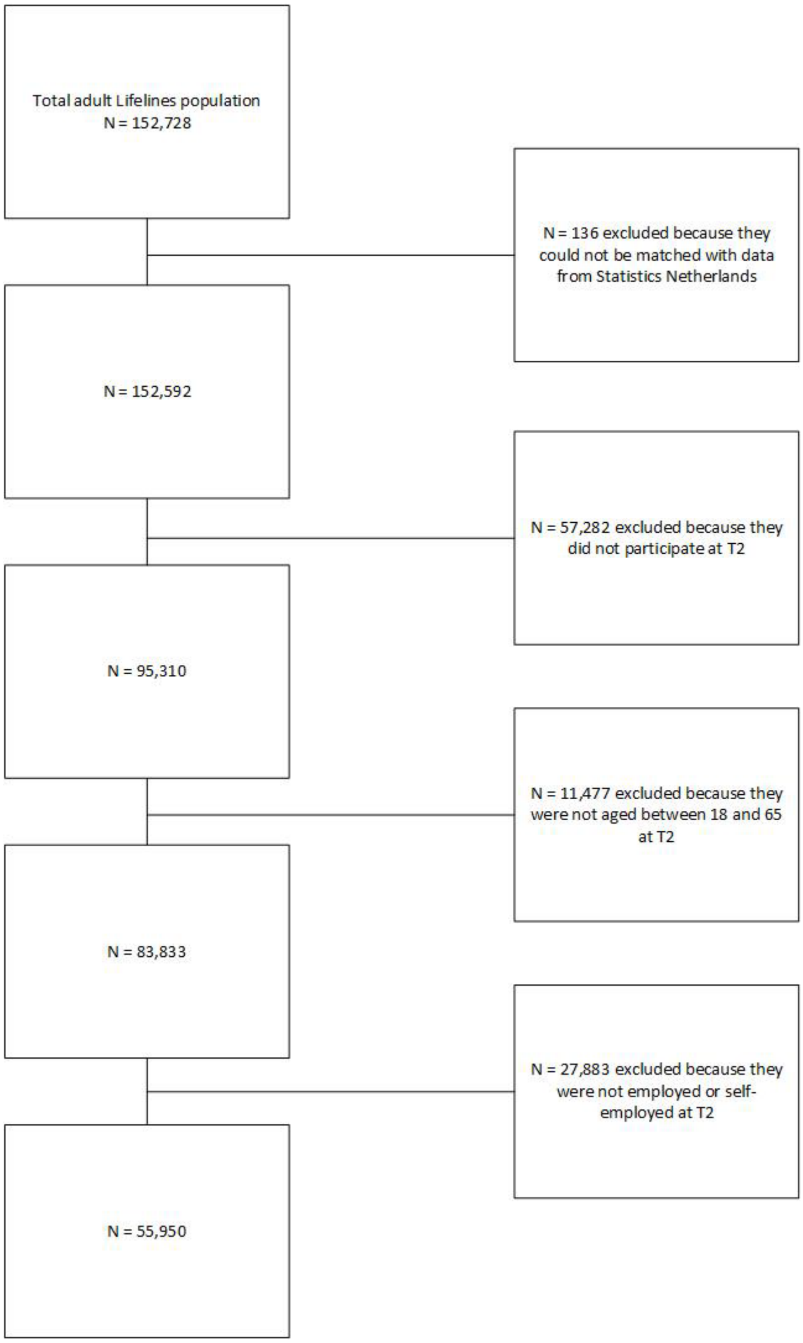

Supplementary table S1. Information on measurements from the Lifelines Cohort Study and Statistics Netherlands

	T0 (2006-2013)	T2 (2012-2015)	Follow-up (T2-2018)
Lifelines Cohort Study	Depression (MINI)	Working conditions: Job demands and job resources (COPSOQ-II)	
Lifelines Cohort Study	Control variables: Educational level, gender	Control variables: Age, perceived health	
Statistics Netherlands			Employment
